# Calidad de vida y funcionalidad en sobrevivientes de cuidados intensivos: Una revisión exploratoria

**DOI:** 10.15649/cuidarte.2269

**Published:** 2023-03-31

**Authors:** Julia Andrea Arias-Díaz, Dulfary Mejía-Vanegas, Lleraldyn Leyton-Toro, Katherine Yuliet Ayala-Grajales, Angie Manuela Becerra-Londoño, Jorge Iván Vallejo-Ospina, Ángela María Rincón-Hurtado

**Affiliations:** 1 . Fundación Universitaria del Área Andina. Pereira, Colombia. Email: dmejia2@areandina.edu.co Autora de correspondencia Fundación Universitaria del Área Andina Fundación Universitaria del Área Andina Colombia dmejia2@areandina.edu.co; 2 . Fundación Universitaria del Área Andina. Pereira, Colombia. Email: jarias77@areandina.edu.co Fundación Universitaria del Área Andina Fundación Universitaria del Área Andina Colombia jarias77@areandina.edu.co; 3 . Fundación Universitaria del Área Andina. Pereira, Colombia. Email: lleyton3@estudiantes.areandina.edu.co Fundación Universitaria del Área Andina Fundación Universitaria del Área Andina Colombia lleyton3@estudiantes.areandina.edu.co; 4 . Fundación Universitaria del Área Andina. Pereira, Colombia. Email: kayala2@estudiantes.areandina.edu.co Fundación Universitaria del Área Andina Fundación Universitaria del Área Andina Colombia kayala2@estudiantes.areandina.edu.co; 5 . Fundación Universitaria del Área Andina. Pereira, Colombia. Email: abecerra18@estudiantes.areandina.edu.co Fundación Universitaria del Área Andina Fundación Universitaria del Área Andina Colombia abecerra18@estudiantes.areandina.edu.co; 6 . Fundación Universitaria del Área Andina. Pereira, Colombia. Email: jvallejo20@estudiantes.areandina.edu.co Fundación Universitaria del Área Andina Fundación Universitaria del Área Andina Colombia jvallejo20@estudiantes.areandina.edu.co; 7 Fundación Universitaria del Área Andina. Pereira, Colombia. Email: amrincon@areandina.edu.co Fundación Universitaria del Área Andina Fundación Universitaria del Área Andina Colombia amrincon@areandina.edu.co

**Keywords:** Cuidados Intensivos, Enfermedad Crítica, Calidad De Vida, Debilidad Muscular, Actividades Cotidianas., Intensive Care, Critical Illness, Quality of Life, Muscle Weakness, Activities of Daily Living., Terapia Intensiva, Doenga Crítica, Qualidade de Vida, Fraqueza Muscular, Atividades de Cotidianas.

## Abstract

**Introducción:**

La calidad de vida en pacientes críticos que sobreviven al tratamiento en unidades de cuidados intensivos es inferior al de la población general. La condición de salud basal y la severidad de la condición clínica al ingreso a terapia intensiva son factores de riesgo para la calidad de vida y la funcionalidad.

**Objetivo:**

Analizar el nivel de conocimiento en la calidad de vida y la funcionalidad de los sobrevivientes de cuidados intensivos.

**Material y métodos:**

Se realizó una revisión exploratoria en las bases de datos: Scielo, PubMed, Science Direct, ProQuest, Redalyc, Dialnet, OVID, Scopus, publicados entre enero del año 2010 y mayo del año 2020. El estudio se desarrolló según la estructura de la Metodología PRISMA. Se revisaron y analizaron los textos completos que cumplían los criterios de inclusión para la selección final de los artículos.

**Resultados:**

De 1814 artículo seleccionados, se eligieron 65 artículos que describen la calidad de vida y la funcionalidad en pacientes después de cuidados intensivos, y finalmente, 16 artículos son incluidos, donde se analizaron las características de los artículos, las características de la población estudiada, y las variables de análisis sobre la evaluación de la calidad de vida y la funcionalidad en los sobrevivientes después cuidados intensivos.

**Conclusiones:**

Los estudios sobre calidad de vida y funcionalidad en sobrevivientes de cuidados intensivos se realizaron en mayor proporción en Europa en los años 2010 a 2016. Con estudios observacionales prospectivos que correlacionan los factores que determinan la salud mental y física después del egreso de cuidados intensivos. Se aplicaron múltiples escalas siendo las más utilizadas SF-36 y el EQ-5D para evaluar la calidad de vida y del índice de Barthel para determinar el estado de funcionalidad en los egresados de cuidados intensivos. El SF-36 y el índice de Barthel reportaron una afectación en la calidad de vida y en la funcionalidad en la población sobreviviente de cuidados intensivos.

## Abstract (en)

**Introduction:**

The quality of life of critically ill patients who survive treatment in intensive care units is lower than that of the general population. Baseline health status and severity of clinical condition on admission to intensive care are risk factors for quality of life and functionality.

**Objective:**

To analyze the level of knowledge on quality of life and functionality of intensive care survivors.

**Materials and methods:**

An exploratory review was conducted by searching studies published between January 2010 and May 2020 in Scielo, PubMed, Science Direct, ProQuest, Redalyc, Dialnet, OVID, and Scopus databases. The study was conducted according to the PRISMA statement. Full texts that met the inclusion criteria were reviewed and analyzed in the final selection of articles.

**Results:**

Of 1814 articles identified, 65 articles describing the quality of life and functionality in post-intensive care patients were screened. Finally, 16 were included to analyze the article's characteristics, population characteristics, and variables of analysis for assessing the quality of life and functionality of post-intensive care survivors.

**Conclusions:**

Studies on quality of life and functionality in intensive care survivors were conducted mainly in Europe between 2010 and 2016. They are primarily prospective observational studies correlating factors determining mental and physical health after intensive care discharge. Multiple scales were applied; the most used were the SF-36 and the EQ-5D to assess the quality of life and the Barthel Index to determine functional status in patients discharged from intensive care. The SF- 36 and Barthel index reported impaired quality of life and functionality of the intensive care survivor population.

## Resumo

**Introdujo:**

A qualidade de vida em pacientes críticos que sobrevivem ao tratamento em unidades de terapia intensiva é inferior a da populagáo geral. A condigáo de saúde de base e a gravidade do quadro clínico na admissáo a terapia intensiva sáo fatores de risco para qualidade de vida e funcionalidade.

**Objetivo:**

Analisar o nível de conhecimento sobre qualidade de vida e funcionalidade de sobreviventes de terapia intensiva.

**Material e métodos:**

Foi realizada uma revisáo exploratória nas seguintes bases de dados: Scielo, PubMed, Science Direct, ProQuest, Redalyc, Dialnet, OVID, Scopus, publicadas entre janeiro de 2010 e maio de 2020. O estudo foi desenvolvido de acordo com a estrutura da Metodologia PRISM. Os textos completos que atenderam aos critérios de inclusáo foram revisados e analisados para a selegáo final dos artigos.

**Resultados:**

Dos 1.814 artigos selecionados, foram escolhidos 65 artigos que descrevem a qualidade de vida e funcionalidade em pacientes após terapia intensiva e, por fim, sáo incluídos 16 artigos, onde sáo apresentadas as características dos artigos, as características da populagáo estudada e as variáveis de análise sobre a avaliagáo da qualidade de vida e funcionalidade em sobreviventes após terapia intensiva.

**Condusdes:**

Estudos sobre qualidade de vida e funcionalidade em sobreviventes de terapia intensiva foram realizados em maior proporgáo na Europa nos anos de 2010 a 2016. Com estudos observacionais prospectivos que correlacionam os fatores que determinam a saúde mental e física após a alta da terapia intensiva. Foram aplicadas múltiplas escalas, sendo as mais utilizadas o SF-36 e o EQ-5D para avaliar a qualidade de vida e o índice de Barthel para determinar o estado de funcionalidade em egressos de terapia intensiva. O SF-36 e o índice de Barthel relataram impacto na qualidade de vida e funcionalidade na populagáo sobrevivente de terapia intensiva.

## Introducción

La evolución ideal de un paciente que ha sufrido un ingreso en una Unidad de Cuidados Intensivos (UCI) consiste en volver a su estado de salud previo, o al esperado para una persona del mismo grupo de edad y situación médica^1^. Una característica importante de la población que se atiende en una UCI, es que está formada por un grupo heterogéneo de pacientes que abarca diferentes patologías que frecuentemente sólo tienen en común el haber sufrido un problema crítico que motiva su ingreso para ser soportado por tecnología avanzada^2^.

Los avances científicos, académicos y técnicos, han permitido que las UCI consigan índices de supervivencia cada vez más altos^3^. Sin embargo, desde una visión integral de la salud y de la continuidad asistencial, la atención a la salud emocional y el seguimiento del estado de funcionalidad después de la supervivencia de UCI, se está planteando como algo totalmente necesario, para asegurar dimensiones positivas en la calidad de vida y de la funcionalidad de esta población^4^.

La calidad de vida en pacientes críticos que sobreviven al tratamiento en una UCI es inferior al de la población general; es decir, la condición de salud y la severidad de la enfermedad al ingreso a UCI son factores determinantes en la calidad de vida^5^. El paciente al egreso de la UCI presenta un deterioro de la independencia funcional y la incapacidad de realizar sus propias tareas llegando a la dependencia temporal o permanente, debido a que la autonomía puede disminuirse o incluso perderse por la presencia de trastornos cognitivos, depresión, estrés postraumático, procesos patológicos crónicos y agudos, siendo estos factores el motivo de la alteración de la calidad de vida en el paciente después de la UCI^6^.

Por lo anterior, se hace necesario realizar acciones para disminuir los impactos sobre el nivel de independencia funcional y la calidad de vida de los pacientes que se ven obligados a estar en terapia intensiva, sometidos a reposo prolongado y /o asistencia con equipos de alta tecnología de manera invasiva, como es el uso de la ventilación mecánica, terapia de reemplazo renal, y el uso de medicamentos como sedantes, antipsicóticos, barbitúricos, anticonvulsivantes, relajantes musculares, cortico-esteroides y analgésicos opioides que impactan en la fuerza muscular de estos pacientes^5^.

Algunos estudios previos han mostrado que al egreso de la UCI hay una disminución significativa en la funcionalidad y fuerza muscular de los pacientes en comparación a la que reportaban antes del estado crítico, observándose secuelas y dificultades para realizar sus actividades de la vida diaria de manera independiente hasta por 12 meses después del alta de UCI^6^.

Por otra parte, otros autores estudiaron la calidad de vida, el delirium, la depresión, el estrés postraumático y los problemas psicológicos con que egresaban los pacientes después del paso por la UCI. Un estudio de cohorte retrospectiva realizado en Brasil durante el 2017 indicó que el 45% de los sobrevivientes de la UCI presentaron alteraciones psicológicas, incluyendo depresión, ansiedad, y la mayoría de los pacientes (84,4%) describió tener algo de memoria de los eventos en la UCI^7^. De este grupo, el 39.1% tuvo recuerdos de eventos reales, y el 45,3% tenía recuerdos de ilusión (solo o en combinación con hechos reales), como sueños (13.3%), pesadillas (7.0%) y alucinaciones (25.0%^7^).

Otro estudio, realizado por Skinner et al en Australia durante el 2011, evaluaron la calidad de vida con el instrumento SF-36 a 67 pacientes al momento del alta de la UCI y 6 meses después encontraron disminuciones significativas tanto en el componente mental como en el componente físico, y a los 6 meses mejoraron esos resultados; pero, no lo suficiente para retornar al estado inicial de antes del paso por la UCI^8^. En otro estudio, realizado por Silveira et al en Brasil en el año 2018, evaluaron la funcionalidad en 249 pacientes tratados con ventilación mecánica, utilizando el índice de Barthel, en donde obtuvieron resultados de independencia total al ingreso de UCI, y de dependencia moderada al egreso^9^.

En cuanto a la calidad de vida de los pacientes que sobreviven a una UCI; se ha encontrado que es inferior a la de la población general. En la actualidad, varios estudios describen las patologías, los días de estancia en unidades de terapia intensiva y la morbi-mortalidad de los pacientes en UCI; pero limitadas investigaciones evalúan la funcionalidad y la calidad de vida después de Cuidado intensivo. Por lo tanto, esta investigación busca concientizar a las instituciones prestadoras de salud, sobre la importancia de realizar un seguimiento a la funcionalidad y la calidad de vida del paciente después de UCI, logrando nuevamente su incorporación en la dimensión social con mínimas afectaciones en su funcionalidad^10^.

Actualmente, existe información limitada de los sobrevivientes de la UCI con respecto a la asociación entre el estado funcional al alta hospitalaria y los eventos adversos posteriores al egreso hospitalario^11^. Los estudios en UCI aún prefieren los resultados a corto plazo basados en la mortalidad como sus resultados primarios, a pesar de autores que afirman que la evaluación del resultado después de la estadía en UCI debe incluir medidas en las dimensiones de calidad de vida; no obstante, solo un pequeño número de estudios de resultados basados en la caracterización del paciente en UCI utilizan estas medidas; el personal médico y profesionales de apoyo no están familiarizados con la interpretación de estos resultados en su práctica^12^. De acuerdo con lo anterior, en esta, revisión exploratoria se analizó el estado actual de conocimiento sobre los factores que afectan la calidad de vida y la funcionalidad de los sobrevivientes de cuidados intensivos.

## Materiales y Métodos

La revisión exploratoria se desarrolló según la estructura de la Metodología PRISMA^13^ , realizando una búsqueda en las bases de datos como Scielo, PubMed, Science Direct, ProQuest, Redalyc, Dialnet, OVID, Scopus, de artículos publicados entre enero del año 2010 y mayo del año 2020, en idioma inglés y español, teniendo en cuenta los términos DeCS y MeSH, y conectores boléanos como AND, y OR por ejemplo * Calidad de vida AND Unidad de cuidados intensivos, *Calidad de vida AND personas con limitaciones físicas, *Personas con limitaciones físicas AND Unidad de cuidados intensivos AND actividades de la vida diaria, *Personas con limitaciones físicas AND Estado crítico AND calidad de vida, haciendo uso de la pregunta PICO (P: pacientes adultos críticos, I: calidad de vida y funcionalidad, Comparación: población general, O: sobrevivientes de cuidados intensivos), para definir los criterios de búsqueda, y finalmente la selección de los artículos relacionados con la evidencia sobre la calidad de vida y la funcionalidad de los pacientes que egresan de la unidad de cuidados intensivos.

### Criterios de Selección

Se estableció la pregunta: ¿Qué se conoce acerca de la calidad de vida y la funcionalidad del paciente crítico después de su egreso de la unidad de cuidado intensivo?, seleccionando los textos que cumplían los criterios de inclusión como: I) estudios publicados en el periodo entre 2010 y 2020, II) estudios con todo tipo de diseños, tanto cuantitativos como cualitativos, III) estudios publicados en inglés y español, IV) Estudios que refieran calidad de vida, funcionalidad, complicaciones y supervivencia de paciente crítico después del egreso de cuidado intensivo, y V) investigaciones con resultados publicados en revistas indexadas. Y, criterios de exclusión: I) estudios que no estén disponibles en texto completo, II) estudios producto de revisión de literatura, III) estudios realizados en cuidado intensivo en población pediátrica o neonatal, IV) estudios de población adulta en áreas de cuidado paliativo en el marco de la enfermedad terminal, y V) tesis de pregrado y posgrados.

### Selección de Artículos

**Figura 1:**
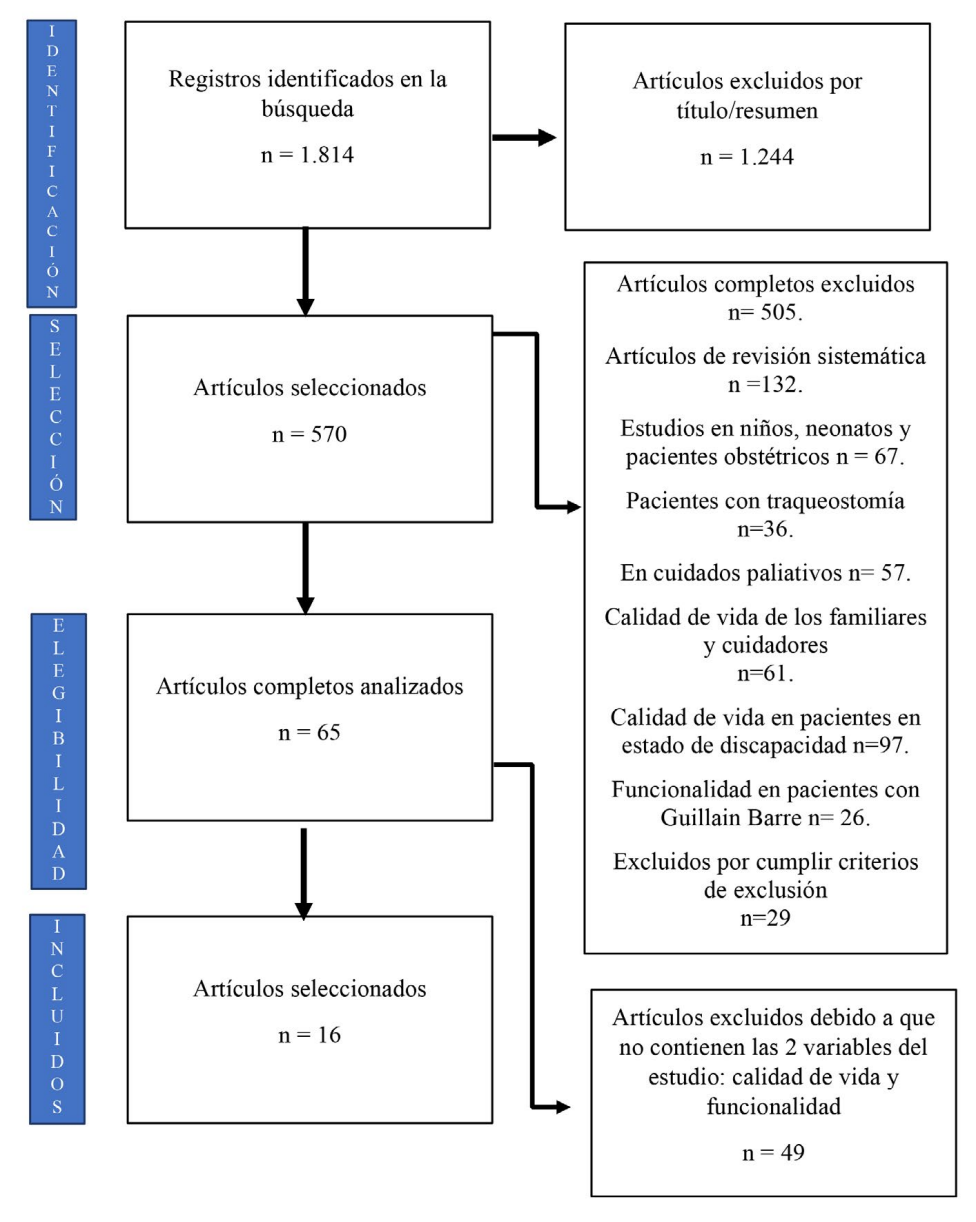
Diagrama de flujo del proceso de selección de estudios para la revisión exploratoria.

El estudio se desarrolló según la Metodología, Preferred reporting items for systematic reviews and meta-analyses (PRISMA), cumpliendo con los criterios de elegibilidad fundamentados en la pregunta PICO (Población: pacientes adultos críticos, Intervención: calidad de vida y funcionalidad, Comparación: población general, O: sobrevivientes de cuidados intensivos). La selección inicial fue de 1814 artículos, de los cuales fueron excluidos 1244 por título y resumen, quedando seleccionados 570; de estos artículos se excluyeron 505 artículos incompletos, 132 artículos de revisión sistemática, 67 estudios que involucraron niños, neonatos y pacientes obstétricos, 36 estudios que incluían pacientes con traqueostomía, 57 estudios con pacientes en cuidados paliativos, 61 artículos que mencionan calidad de vida de los familiares y cuidadores, así como 97 estudios sobre calidad de vida en pacientes en estado de discapacidad, 26 artículos que relacionaban la funcionalidad con pacientes diagnosticados con Guillain Barré, y 29 excluidos por cumplir criterios de exclusión, resultando elegidos 65 artículos de texto completo; y finalmente, son incluidos 16 artículos, debido a que incluyen las 2 variables de estudio, calidad de vida y funcionalidad, en pacientes después de cuidados intensivos. (Figura 1)

### Extracción de síntesis de datos

Los 16 artículos incluidos fueron revisados y analizados por los investigadores quienes realizaron una base de datos con la información más relevante para la investigación como: Autor, año, lugar de publicación, tipo de estudio, número de participantes, género, edad, diagnóstico médico de ingreso, APACHE (ACUTE PHYSIOLOGY SCORE AND CHRONIC HEALTH EVALUATION), número de días en hospitalización, días en UCI, días de ventilación mecánica, mortalidad, escalas y/o test de medida y puntuación sobre calidad de vida y Funcionalidad. Posterior a esto, esta información fue agrupada según las características de artículos, las características de la población estudio, y los datos sobre la valoración de la calidad de vida y la funcionalidad de los sobrevivientes después de la UCI.

### Riesgo de sesgo en los estudios

Se incluyeron los artículos que cumplen con los criterios de inclusión y exclusión para minimizar el sesgo de selección de la información.

### Calidad de los artículos

En la evaluación de la calidad metodológica de los artículos se utilizó la declaración de STROBE (Tabla 1), para los estudios observacionales y la escala de PEDro para los ECA. En STROBE se obtuvo una calificación de 20 puntos para 10 artículos (62%) 18 puntos para 3 artículos (19%) y 17 puntos para el resto de los artículos (19%). Mientras que, con la escala de PEDro, se evalúo 1 articulo, obteniendo puntuación de 10.

### Consideraciones éticas

Este estudio corresponde a una revisión exploratoria de la literatura, no fue sometido a aval del Comité de Ética; pero, sí se tuvo en cuenta la normatividad correspondiente a derechos de autor, lo que hace que los autores garanticen la originalidad del texto completo.

## Resultados

Para la revisión exploratoria se incluyeron 16 artículos (Figura 1), los cuales contenían información de las dos variables de interés en el estudio, para posterior análisis de los datos sobre calidad de vida y funcionalidad del paciente crítico después de su egreso de cuidado intensivo.

En la Tabla 1, se muestran los 16 artículos incluidos para la revisión de la calidad de vida y la funcionalidad del paciente crítico después de su egreso de cuidado intensivo, la mayor proporción se encontró en los años (Gráfico 1) 2011 (3) y 2015 (3), seguido de los años 2016 (2), 2018 (2), 2019 (2) y 2020 (2), y en 2014 (1) y 2017 (1). Los estudios fueron realizados en su mayoría en España 18,7% (3), seguido de Australia 12,5% (2), Argentina 12,5% (2) y Suiza 12,5% (2), Canadá 6,25% (1), EEUU 6,25% (1), Brasil 6,25% (1), Finlandia 6,25% (1), Grecia 6,25% (1), Holanda 6,25% (1) y Francia 6,25% (1). (Gráfico 2)

**Gráfico 1:**
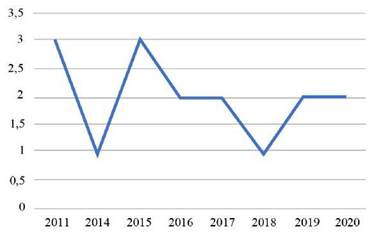
Cantidad de estudios encontrados por año.

**Gráfico 2:**
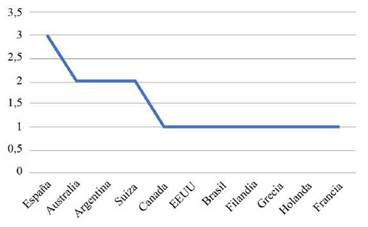
Cantidad de estudios realizados por lugar de publicación.

El 94% (15) de estos estudios tienen diseño metodológico observacional prospectivo y el 6% (1) ensayo aleatorio controlado (Tabla 1). El 100% de los estudios analizados realizaron seguimientos a los sujetos, el 32,4% a los 6 meses, el 20,6% a los 12 meses, el 17,6% a los 3 meses, 17,6% antes de los 3 meses y el 11,8% después de los 12 meses. (Gráfica 3). En cuanto al número de seguimientos que realizaron dentro de los estudios a los pacientes después de su estadía en UCI, se evidenció que el 43,8% lo realizó al menos 2 veces, el 25% una vez, el 25% tres (3) veces y el 6,3% lo realizó 4 veces. (Gráfica 4).

**Gráfico 3:**
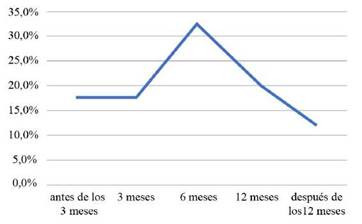
Tiempo de los seguimientos.

**Gráfico 4:**
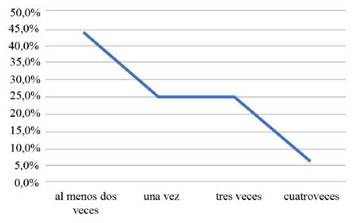
Cantidad de los seguimientos.

**Tabla 1:** Calidad de vida y Funcionalidad del paciente crítico después de su egreso de cuidado intensivo.

Autor	Año	Lugar	Diseño Metodológico	PE	Spost UCI	Score Calidad vida	Score Funcionalidad	Score (Validación)
Elliott, et al^9^	2011	Australia	Ensayo controlado aleatorio	195	1, 8, 26 semanas después de UCI	SF 36	6MWT	10 PEDro
Sacanella, et al10	2011	España	Observacional prospectivo	112	3,6, 12 meses después de UCI	EQ-5D	Barthel Lawton	20 Strobe
Vest, et al^14^	2011	EEUU	Cohorte Prospectivo	309	1 mes y al año después de UCI	SF-12	KATZ	20 Strobe
Busico, et al^15^	2014	Argentina	Observacional prospectivo	181	4, 12 meses después de UCI	EQ-5D	Barthel	17 Strobe
Goixart, et al^16^	2015	España	Observacional prospectivo	110	6 y 12 meses después de UCI	SF 36 EQ-5D	SF 36	18 Strobe
Das Neves, et al17	2015	Argentina	Cohorte Prospectivo	112	1,3,6,12 meses después de UCI	EQ-5D	6MWT	20 Strobe
Feemster, et al18	2015	Canadá	Longitudinal prospectivo	668	12, 24, 30 meses después de UCI	SF-36	SF-36	20 Strobe
Sharon, et al^9^	2016	Australia	Observacional prospectivo	193	2,6 meses después de alta UCI	SF-36	SF-36 ICEQ	20 Strobe
Villa, et al^19^	2016	Madrid	Observacional Prospectivo	176	3, 6 y 12 meses después de UCI	SF-36	Barthel	20 Strobe
Berkius, et al^20^	2017	Suiza	Prospectivo longitudinal	31	6,12,24 meses después de UCI	EQ-5D SF-36	SF-36	17 Strobe
Silveira Vesz, et al^21^	2018	Brasil	Observacional Prospectivo	160	1 semana después de alta UCI	WHo- qoL-Bref	Barthel	17 Strobe
Ferrand, et al^22^	2018	Francia	Observacional prospectivo	438	3 y 6 meses después del alta de UCI	SF-36	NR	18 Strobe
Sidiras, et al^23^	2019	Grecia	Prospectivo	128	Alta cuidados intensivos, 3, 6 meses después UCI	SF-36	FIM	20 Strobe
Niittyvuopio, et al ^24^	2019	Finlandia	Prospectivo Longitudinal	332	5 años después de alta UCI	SF-36	RAND-36	20 Strobe
Beumeler, et al25	2020	Holanda	Prospectivo longitudinal	120	Alta cuidados intensivos, 3,6 meses después de UCI	RAND-36 SF-36	Barthel	20 Strobe
Eggmann, et al26	2020	Suiza	Cohorte Prospectivo	115	Alta hospitalaria, 6 meses después de UCI	SF-36	MRC-SS FIM	18 Strobe

* PE: Población Estudio. SPost UCI: Seguimiento después de la UCI. UCI: Unidad de cuidado intensivo. MWT: Metros recorridos. EPOC: Enfermedad Pulmonar Obstructiva Crónica. TEC: Trauma Craneoencefálico. SDRA: Síndrome de Distrés Respiratorio Agudo.

En la Tabla 2, se describen las características de la población estudio revisadas en cada artículo incluido. La población total en promedio es de 191 participantes, teniendo en cuenta que un estudio reportó 668 participantes, mientras que otro 31. En promedio 64,8 son de género femenino (F), mientras que el 86,7 pertenecen al género masculino (M). El promedio de edad es de 62,3 años, con 9,2 días en promedio de estancia en UCI, 9,5 días de ventilación mecánica, 12,2 días de hospitalización, y el puntaje de evaluación de la gravedad al ingreso a la unidad de cuidado intensivo fue dado por la escala APACHE de 14,8. Los diagnósticos de ingreso a la UCI reportados por los autores, describieron causas Respiratorias 35%, Neurológicas 18%, Cardiovasculares 15%, Gastrointestinales 11%, Trauma 11%, Renal 3%, Hepática 1%, Otras causas 6% como Diabetes, Emergencia médica, Infección y Otras cirugías.

**Tabla 2:** Características de la población evaluadas.

Autor	PE	Género		Edad	Días UCI	Días VM	Días Hops	Diag. Ingreso UCI	APACHE
		F	M						
Elliott, et al^9^	195	76	119	57	6	91	18,5	Gastrointestinales, Respiratorios y Cardiovascular, Sepsis y Trauma.	19,1
Sacanella, et al10	112	43	57	73,4	9.1	0	0	Enfermedad respiratoria, Sepsis grave/ shock séptico, Enfermedad cardíaca, Enfermedad cerebrovascular.	19,1
Vest, et al ^14^	309	164	145	74,7	11	6	0	Respiratoria, Sepsis, Gastrointestinal	23
Busico, et al15	77	41	36	65	9	4	0	IRA, Enfermedad neuromuscular, EPOC, coma	NR
Goixart, et al16	110	27	83	43,5	8	0	20	TCE, trauma Tórax, trauma Abdomen, trauma Extremidades	10
Das Neves, et al^17^	112	36	76	33	21	15	47	Shock, SDRA, Trauma, Renal, Emergencia Médica	15
Feemster, et al18	668	19	649	66,8	11	0	0	Sepsis,SDRA, Falla Multiorganica	NR
Villa, et al^19^	176	88	88	81	4	4	11	Cardiovascular, Infección, Respiratorio, Neurológico	19,4
Berkius, et al20	31	12	19	69,7	2,2	0	10	EPOC	19,5
Silveira Vesz, et al^21^	160	68	92	71,5	8,9	0	0	NR	20,1
Ferrand, et al22	220	63,8	156,2	63	6	1	0	SDRA, Fármacos inotrópicos, Terapia de reemplazo renal, Transfusión de sangre.	NR
Sidiras Georgios, et al23	128	45	83	58	26	18	25	Neurológico respiratoria sepsis, Trauma, Gastrointestinal	18
Niittyvuo- pio, et al^24^	332	123	209	49	7,4	0	23	Sepsis, Gastrointestinal, Neumonia, TEC Hemorragia Cerebral	18
Beumeler, et al25	120	94	26	61	7	3,8	22	Cardiaca, Neumonía, Enfermedad Hepatica, septisemia.	20
Eggmann,	115	72	43	67,5	1,7	0	5,93	IRA, Cirugía Cardíaca, Insuficiencia hemodinamica, Gastroenterología, Otras cirugías, Neurología/Neurocirugia, Trauma	21

*PE: Población Estudio. UCI: Unidad de Cuidados Intensivos. VM: Ventilación Mecánica. Días Hosp: Días de hospitalización. Diag. Ingreso UCI: Diagnóstico médico de ingreso a la UCI. APACHE: ACUTE PHYSIOLOGY SCORE AND CHRONIC HEALTH EVALUATION. TCE: Trauma Craneoencefálico. SDRA: Síndrome de Distrés Respiratorio Agudo. IRA: Insuficiencia respiratoria Aguda. **NR: No Reporta

En la Tabla 3, se registraron los datos obtenidos de la revisión de los estudios, sobre la evaluación de la calidad de vida de los pacientes después de su egreso de las unidades de cuidado intensivo. En los 16 artículos evaluaron la calidad de vida y realizaron seguimientos a los sujetos que participaron en las investigaciones. Los instrumentos de medición reportados fueron el SF-36, EQ-5D, SF-12 y Whoqol-Bref en la evaluación de los dominios físico y mental de la población sobreviviente de terapia intensiva. (Tabla 1)

Analizando los resultados del dominio físico todos los valores estuvieron por debajo del rango esperado, lo que muestra una disminución de la calidad de vida comparada con la población normal. En el componente mental algunos autores informan una buena condición en ese dominio. Un artículo^14^, evalúo la calidad de vida con el SF-12 (versión corta del SF-36), el resultado de la puntuación en los dominios mental y físico reportaron una disminución en la calidad de vida al igual que los análisis del SF-36. Las evaluaciones realizadas con la escala EQ-5D^10^^,^^17^^,^^15^, reportaron una mejor calidad de vida en los sobrevivientes de cuidados intensivos con una condición basal cercana a la de la población normal.

Por el contrario, la escala Whoqol-Bref^21^ tuvo puntuaciones de salud mental y física disminuidas relacionando una significativa afectación de la calidad de vida en la población sobreviviente de cuidados intensivos. En los estudios evaluados, reportaron mayor compromiso en la salud física que en la salud mental^27^. Los factores relacionados con el deterioro del componente físico son la edad, las comorbilidades, los días de ventilación mecánica, los días de estancia en cuidados intensivos y la condición basal de salud antes del ingreso a unidad de cuidados intensivos.

**Tabla 3:** Calidad de vida de los pacientes después de su egreso de las unidades de cuidado intensivo.

Primer Autor							
	SF 36			SF- 12		EQ-5D HRQOL	WHOQoL-Bref
	Físico	Mental	Nottingham Health profile HRQOL	Físico	Mental		
Elliott, et al^9^	42,9	47,2	------	------	-------	-------	-------
Sacanella, et al^10^	------	------	------	------	-------	76,1	-------
Beumeler, et al^25^	46,7	48,8	------	------	-------	-------	44
Goixart, et al^16^	44,6	40,9	------	------	-------	45,4	-------
Ferrand, et al^22^	44	43	------	------	-------	-------	-------
Silveira Vesz, et al^21^	------	------	------	------	-------	-------	14
Berkius, et al^20^	68,2	45,5	------	------	-------	43	-------
Niittyvuopio, et al^24^	60	76	------	------	-------	-------	-------
Sidiras Georgios, et al^23^	48	56	79	------	-------	-------	-------
Vest, et al^14^	-------	-------	-------	37,2	51,5	-------	-------
Feemster, et al^18^	35	47,3	-------	-------	-------	-------	-------
Villa, et al^19^	62,4	66,6	-------	-------	-------	-------	-------
Das Neves, et al^17^	-------	-------	-------	-------	-------	74	-------
Eggmann, et al^26^	42,6	76	-------	-------	-------	-------	-------
Busico, et al^15^	-------	-------	-------	-------	-------	69	-------
Sharon, et al ^9^	32,7	39,8	-------	-------	-------	-------	-------

En Tabla 4, se registraron los datos obtenidos de la revisión de los estudios sobre la evaluación de la funcionalidad o estado funcional del paciente crítico después de su egreso de cuidado intensivo, de los 16 artículos incluidos y analizados, 9 reportaron resultados sobre la valoración de la funcionalidad. Las pruebas o medidas utilizadas dentro de los estudios fueron heterogéneas, encontrando las siguientes: Barthel (31,25%), FIM (12,5%), KATZ (6,25%) y Lawton (6,25%).

Las evaluaciones realizadas con el índice de Barthel^10^^,^^25^^,^^21^^,^^19^^,^^15^ reportaron resultados de 96,4 (Dependencia leve), 90, 87, 85 y 87,5 (Dependencia moderada); con la escala FIM^23^^,^^26^, reportaron resultados de 65 (Dependencia) y 36 puntos (Dependencia completa); con el índice de Katz^14^ obtuvieron una calificación de H (Dependiente en al menos 2 actividades de la vida diaria); y con escala de Lawton^10^ reportaron 6,8 puntos (dependencia moderada en las actividades de la vida diaria). Cabe mencionar que algunos autores toman como referencia otros test o medidas que les permite asociar los resultados con el estado funcional de los pacientes, como la Escala MRC para fuerza muscular^26^, Dinamometría para fuerza muscular de agarre^25^, test de la caminata de los 6 minutos^16^^,^^26^, test timed "Up &Go"^26^, Escala de Berg^16^, Índice de movilidad de Morton^25^, e incluso SF-36 componente físico^18^.

**Tabla 4:** Estado Funcional del paciente crítico después de su egreso de cuidado intensivo.

Primer Autor		Funcionalidad		
	Barthel	FIM	Katz	Lawton
Sacanella, et al^10^	96,4	-------	-------	6,8
Beumeler, et al^25^	90	-------	-------	-------
Goixart, et al^16^	-------	-------	-------	-------
Silveira Vesz, et al^21^	87	-------	-------	-------
Sidiras, et al^23^	-------	65	-------	-------
Vest, et al^14^	-------	-------	H	-------
Villa, et al^19^	85	-------	-------	-------
Eggmann, et al^26^	-------	36	-------	-------
Busico, et al^15^	87,5	-------	-------	-------

* FIM: Functional Independence Measure (Medición de la Independencia Funcional).

## Discusión

Los aportes de esta revisión fue la identificación y análisis de la literatura con evidencia, donde se relacionaba el nivel de funcionalidad basal, el diagnóstico, la edad, el género y los días de estancia en terapia intensiva, como factores que condicionaban la calidad de vida y la funcionalidad después del egreso de la unidad de cuidados intensivos.

Los autores utilizaron diferentes escalas o instrumentos de medición para cada una de las variables. Las escalas para valorar calidad de vida encontradas en los estudios fueron: el SF-36 (68%), EQ-5D (EuroQol) (19%), SF-12 (6%), Whoqol-Bref (6%); y para funcionalidad o estado funcional fueron: Barthel (31,25%), FIM (12,5%), KATZ (6,25%) y Lawton (6,25%). Los autores aplicaron 8 escalas para evaluar la calidad de vida y la funcionalidad en la población sobreviviente de cuidados intensivos.

Todos los instrumentos utilizados son escalas genéricas utilizadas y validadas en diferentes poblaciones para determinar la calidad de vida relacionada con la salud, y para la funcionalidad; sin embargo, las escalas de uso más frecuente entre los estudios fueron la SF-36^27^ y el EQ-5D^30^, para evaluar la calidad de vida, y el índice de Barthel^31^, para determinar el estado de funcionalidad en los egresados de cuidados intensivos.

Las evaluaciones realizadas con la escala EQ-5D^10^^,^^17^^,^^15^ reportaron una mejor calidad de vida en los sobrevivientes de cuidados intensivos con una condición basal cercana a la de la población normal, estos resultados se relacionan con lo encontrado por Peter Schenk et al en el año 2012^32^^), (^^33^, donde describieron que la puntuación de la calidad de vida relacionada con la salud no cambió significativamente a lo largo del tiempo en los sobrevivientes de la UCI, utilizando como escala de medición el EQ-5D.

Los autores reportaron que la población sobreviviente de enfermedades críticas y egresados de cuidados intensivos tenían un grado de dependencia moderada a severa; algunos autores lo relacionaban a las deficiencia en fuerza muscular, equilibrio y estados de hipermetabolismo (sepsis, SDRA, trauma^34)^. También, reportaron que algunos sujetos lograron mejorar su grado de independencia a los 6 meses de su alta de cuidados intensivos; sin embargo, registraron que no logran la independencia a su estado basal antes de la patología crítica^35^.

Analizando los resultados en el dominio de salud física de los hallazgos, estuvieron por debajo del puntaje esperado, lo que muestra una disminución de la calidad de vida comparada con la población normal^36^. El deterioro físico estaba asociado a estancia prolongada en UCI, comorbilidades, requerimientos altos de dosis de vasopresores, días de ventilación mecánica y presencia de debilidad muscular. El deterioro mental^37^ se asociaba a la presencia de delirium, administración de sedación profunda (Haloperidol, propofol, fenitoina,benzodiacepinas) y ausencia de visita de familiares y menor atención de enfermería^24^. En lo estudiado hasta el momento; durante la permanencia en cuidados intensivos los pacientes experimentan la presencia real de factores de estrés vinculados con amenaza de muerte, pensamiento aterrador, trastornos del sueño, pérdida de control de su entorno personal y familiar, pérdida de autonomía, abandono de roles individuales, familiares y sociales, aislamiento familiar y social, temor a la discapacidad o miedo a los tratamientos invasivos.

Los estudios de esta revisión analizaron otras variables relacionadas con peor calidad de vida del paciente críticamente enfermo después de su egreso de cuidados intensivos: tener una edad mayor de 45 años, peor calidad de vida previa, comorbilidades y haber sufrido una lesión grave tipo trauma^16^. La duración de la ventilación mecánica disminuye el estado físico funcional después del alta de la UCI en los sobrevivientes^21^. Otros factores modificables durante y después de una estancia en la unidad de cuidados intensivos, incluyen las alteraciones del sueño, la depresión y el estrés post-traumático^27^. Estos hallazgos se asemejan a lo reportado por otros autores^2^^,^^38^^,^^39^, que tuvieron como resultados una calidad de vida significativamente más baja al cabo de un año en comparación con la población general y significativamente reducida en comparación con sus estados antes de la admisión a la unidad de cuidados intensivos.

Así mismo el autor reporta que el control del estrés pos-traumático, el optimismo y la capacidad de afrontamiento de los sujetos, fueron predictores de una mejor calidad de vida y la posibilidad de regreso a las actividades de desarrollo habitual. En un trabajo^8^ sobre la experiencia de adultos críticamente enfermos hospitalizados en UCI significó una motivación para cambiar la forma de vivir y de pensar, permitiendo avanzar en el aprendizaje y en el crecimiento personal, lo que sirvió como base para tomar decisiones respecto a la vida futura. Los pacientes con trauma tuvieron una mayor disminución en las puntuaciones de los dominios físico y mental en el momento de evaluar la calidad de vida. Solo la mitad de los pacientes habían regresado a sus actividades desarrolladas, antes de su ingreso a terapia intensiva^2^^,^^39^.

En los estudios revisados describieron una disminución significativa en la autonomía funcional y la calidad de vida de los sujetos en comparación con el estado basal del paciente^10^, fue evidente que un mayor tiempo de estancia en la unidad de cuidado intensivo podría tener efectos deletéreos sobre la movilidad articular, la fuerza muscular y la capacidad funcional^34^; originando alteraciones en todos los dominios de calidad de vida y dependencia en las actividades de la vida diaria^40^, con un deterioro significativo en la calidad de vida en las dimensiones físicas y mental evaluadas. A los 6 meses de haber abandonado la unidad de cuidados intensivos el componente mental disminuyó más que el componente físico; pero, al egreso de cuidados intensivos se describieron alteraciones más marcadas en la salud física de la población.

Las comorbilidades, la edad y el funcionamiento físico reducido a los 3 meses se identificaron como factores de riesgo para la no recuperación física a largo plazo con afectación en la calidad de vida^25^. Los sobrevivientes de UCI debían ser apoyados en actividades de la vida diaria^14^, consecuencia al deterioro significativo en el estado funcional y aunque se recuperaron modestamente durante el año siguiente, nunca recuperaron su estado inicial^19^, estos mismos datos fueron planteados, describiendo que la discapacidad funcional pre-UCI en actividades básicas, instrumentales y de movilidad se asocia con una mayor mortalidad en el año siguiente al egreso de cuidados intensivos^32^.

La discapacidad prevaleció 6 meses después de la enfermedad crítica en los sobrevivientes de cuidados intensivos y se asoció con una reducción de la calidad de vida^41^. Los pacientes con debilidad muscular adquirida en UCI presentaron una reducción significativa de la fuerza muscular, recuperando los valores normales a los 6 meses del alta hospitalaria^23^. La debilidad muscular al alta de la UCI se asoció con una discapacidad funcional a corto plazo y una estadía hospitalaria prolongada; pero, no con la calidad de vida^26^^,^^42^^,^^43^.

Los determinantes de la calidad de vida y la funcionalidad en sujetos después del alta de cuidados intensivos se relacionaron tanto con las secuelas tardías de la enfermedad crítica, como con las complicaciones que ocurrieron durante la permanencia en cuidado crítico, la edad, el estado basal de salud antes de la patología crítica, las comorbilidades, los días de ventilación mecánica y la estancia prolongada en terapia intensiva^18^. A pesar de la alta carga de síntomas, los sujetos adultos mayores sobrevivientes de cuidados intensivos aún percibían su calidad de vida como buena, por el contrario, los adultos jóvenes vieron significativamente afectada su calidad de vida con compromiso en su salud física y mental^17^.

## Conclusiones

La literatura revisada reporta diferentes investigaciones que evidencia una afectación importante en la calidad de vida y la funcionalidad después del alta de cuidados intensivos. Los instrumentos genéricos utilizados demuestran un buen nivel metodológico en la evaluación de los dominios físicos y mental de la población críticamente enferma. El SF- 36 y el índice de Barthel fueron reportados por los autores como las escalas más utilizadas para evaluar la afectación en la calidad de vida y en la funcionalidad en la población sobreviviente de cuidados intensivos. El desafío futuro para los profesionales del cuidado crítico es implementar estrategias que permitan el seguimiento de la condición física, emocional y social de los sujetos después que abandonan las unidades de cuidados intensivos, para determinar el impacto en su estado de funcionalidad y calidad de vida.
